# Phosphodiesterases and cAMP Pathway in Pituitary Diseases

**DOI:** 10.3389/fendo.2019.00141

**Published:** 2019-03-19

**Authors:** Mariana Ferreira Bizzi, Graeme B. Bolger, Márta Korbonits, Antonio Ribeiro-Oliveira Jr.

**Affiliations:** ^1^Department of Internal Medicine, Federal University of Minas Gerais, Belo Horizonte, Brazil; ^2^Department of Medicine, University of Alabama at Birmingham, Birmingham, AL, United States; ^3^Department of Pharmacology, University of Alabama at Birmingham, Birmingham, AL, United States; ^4^Center for Endocrinology, Barts and The London School of Medicine, William Harvey Research Institute, Queen Mary University of London, London, United Kingdom

**Keywords:** phosphodiesterases, cAMP pathway, pituitary, AIP (Aryl hydrocarbon receptor interacting protein), acromegaly, gigantism

## Abstract

Human phosphodiesterases (PDEs) comprise a complex superfamily of enzymes derived from 24 genes separated into 11 PDE gene families (PDEs 1–11), expressed in different tissues and cells, including heart and brain. The isoforms PDE4, PDE7, and PDE8 are specific for the second messenger cAMP, which is responsible for mediating diverse physiological actions involving different hormones and neurotransmitters. The cAMP pathway plays an important role in the development and function of endocrine tissues while phosphodiesterases are responsible for ensuring the appropriate intensity of the actions of this pathway by hydrolyzing cAMP to its inactive form 5'-AMP. PDE1, PDE2, PDE4, and PDE11A are highly expressed in the pituitary, and overexpression of some PDE4 isoforms have been demonstrated in different pituitary adenoma subtypes. This observed over-expression in pituitary adenomas, although of unknown etiology, has been considered a compensatory response to tumorigenesis. PDE4A4/5 has a unique interaction with the co-chaperone aryl hydrocarbon receptor-interacting protein (AIP), a protein implicated in somatotroph tumorigenesis via germline loss-of-function mutations. Based on the association of low PDE4A4 expression with germline *AIP*-mutation-positive samples, the available data suggest that lack of AIP hinders the upregulation of PDE4A4 protein seen in sporadic somatotrophinomas. This unique disturbance of the cAMP-PDE pathway observed in the majority of *AIP*-mutation positive adenomas could contribute to their well-described poor response to somatostatin analogs and may support a role in tumorigenesis.

## Introduction

Human phosphodiesterases (PDEs) comprise a complex superfamily of enzymes classified into 11 families, encoded by 24 genes representing over 100 different proteins. Many of these genes express several different mRNAs, and the resulting proteins vary widely in their distribution in various tissues and in various intracellular compartments (1).

PDE isoforms differ in their kinetics, distribution, and susceptibility to pharmacological inhibition, as well as selectivity for their different substrates, 3',5' cyclic monophosphate (cAMP) and 3',5' cyclic guanosine monophosphate (cGMP) (1). PDEs share some common structural characteristics: all PDE isoforms have a conserved catalytic domain of ~300 amino acids, located in the C-terminal portion of the protein, and most PDE isoforms contain family-specific regulatory regions in their N-terminal portions (2).

The catalytic regions of each family member differ in amino acid sequence and tertiary structure, which accounts for their specificity for substrate (cAMP and/or cGMP) and their ability to be inhibited by family- and isoform-specific inhibitors. PDE 4, 7, and 8 selectively hydrolyze cAMP, PDE 5, 6, and 9 are selective for cGMP, while PDEs 1, 2, 3, 10, and 11 hydrolyze both, although the specificity is variable (1, 3, 4).

The expression pattern of PDE isoforms varies between tissues and reflects their proliferative state and hormonal stimuli. In this mini review we aim to highlight the important role of these enzymes in pituitary diseases, especially the PDE4A4/5 isoform, encoded by the PDE4A gene, which has been implicated in GH-secreting adenomas due to its selective interaction with aryl hydrocarbon receptor-interacting protein (AIP), a known tumor suppressor gene (5).

## PDEs and cAMP Pathways in the Normal Pituitary Gland

**Figure 1:**
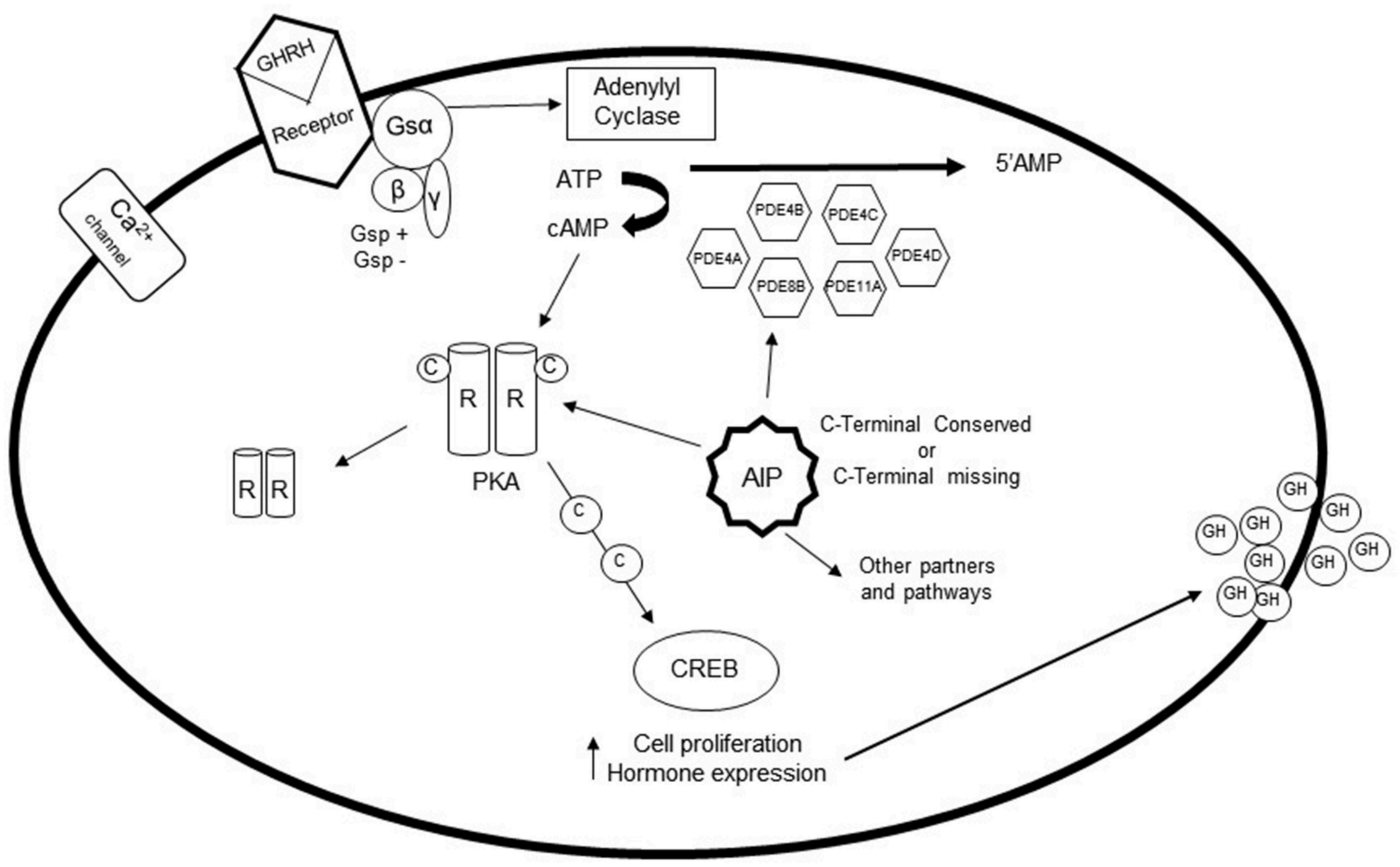
The role of phosphodiesterases (PDEs) in the pituitary gland: After stimulation of somatotroph cells via GHRH, the G protein coupled receptor is activated, which causes a conformational change of the receptor. The Gsα subunit detaches from the complex, and binds to adenylyl cyclase, which catalyzes the conversion of ATP to cAMP. Elevation of intracellular cAMP leads to dissociation of the catalytic subunit and the regulatory subunit of protein kinase A (PKA). Activation of protein kinase A can then phosphorylate a number of targets that regulate effector enzymes and ion channels as well as activates gene transcription that play a role in cell growth and differentiation. PDEs are fundamental in regulating this pathway, since they are the only enzymes capable of hydrolyzing cAMP to its inactive 5'-AMP form. PDE4A, PDE4B, PDE4C, PDE4D, PDE8B, and PDE11A are increased in GH-secreting adenomas, possibly as a compensatory mechanism. However, *gsp* and *AIP* mutations interfere with the expression of these PDEs.

The pituitary gland is a target of different neuroendocrine hormones, which play a crucial role in the control of cell differentiation and proliferation, in addition to hormone secretion, through specific interactions with members of the superfamily of G protein-coupled receptors (GPCRs) (6, 7) (Figure 1). The regulatory, usually hypothalamic, hormone couples to the G protein-coupled receptor in the cell of interest and a conformational change results in activation of the G protein complex. In the case of GHRH, the Gsα subunit is released from the α*βγ* G protein complex and binds to adenylyl cyclase, which then catalyzes the conversion of ATP into the second messenger cAMP. cAMP activates a cascade of other enzymes, thus amplifying the cellular reaction (3). Following GHRH activation of somatotrophs cAMP binds the regulatory subunit of protein kinase A (PKA) (3, 6). The activated catalytic subunit of PKA then phosphorylate a series of targets that regulate effector enzymes, ion channels, and activate the transcription of specific genes that mediate cell growth and differentiation. Additional effectors of cAMP include the exchange factor regulated by cAMP (EPAC) protein, cyclic nucleotide-gated ion channels, Popeye proteins, and possibly additional targets that are still under investigation (1, 8).

PDEs act as regulators of the cAMP pathway, as they are capable of hydrolyzing cAMP to its inactive 5'-AMP form, which is the main pathway for inactivation of cAMP (3, 6). As a consequence, cAMP can either suppress cell proliferation and the mitogenic action of growth factors in some cell types, or conversely, promote the transition from cell cycle G0 to G1 and stimulate cell growth in others (9, 10). It is unclear, for example, why cAMP has a proliferative role in the somatotroph cells while an anti-proliferative role in gonadotroph cells (6, 9, 10). cAMP signaling is temporally, spatially, and functionally regulated by compartmentalization and influenced by a complex network of cell- and tissue-specific downstream effectors and regulators (11). In the pituitary, cAMP acts as a key signaling molecule that controls responsiveness to mitogens and secretagogues, such as hypothalamic hormones, neurotransmitters, and other peripheral factors (7) and a dysregulated cAMP-pathway is involved in the pathogenesis and response to therapy of pituitary adenomas (11).

PDEs are directly implicated in various endocrine disorders affecting the pituitary, adrenals, thyroid, testes, and ovaries (3). Little is known about the expression of PDE isoforms in the pituitary gland, especially in humans, since the vast majority of studies on the association between PDEs and endocrine functions have been performed *in vitro* or in animals. mRNA studies have implicated PDE1, PDE2, PDE4, and PDE11A as being the most highly expressed PDEs in the pituitary (3, 12–14). Interestingly, PDE4 is the only selective PDE for cAMP. The discovery of the physiological role of PDEs in the human pituitary has been hindered due to the lack of availability of specific antibodies. In addition, mRNA does not always reflect the protein amount or function due to variations in translation, protein stability, or posttranslational modifications.

PDE4 isoforms in mammals are encoded by four different genes (PDE4A, PDE4B, PDE4C, and PDE4D) and each of these genes encodes multiple isoforms, through the use of specific promoters for each isoform and alternative messenger RNA processing (15–17). PDE4s differ from the other PDE families by their specific catalytic regions (15–17), as well as by the presence of two “signature” regions called upstream conserved regions (UCRs), which are located in the N-terminal third of the proteins and referred to as UCR1 and UCR2 (18). The various isoforms encoded by each of the PDE4A, PDE4B, PDE4C, and PDE4D genes are divided into three groups: 'long' isoforms that contain both UCR1 and UCR2, “short” isoforms that do not have UCR1 but include UCR2, and “super short” isoforms that do not have UCR1 and contain a truncated UCR2 (18).

PDE4A8 is a long isoform of the PDE4A family with an N-terminal region distinct from the other PDE4A long isoforms PDE4A4, PDE4A10, and PDE4A11 (19–22). It is expressed at significant levels in various regions of the brain, especially in regions involved in coordination, sensation and higher cognitive functions (12, 20). It is also expressed in the pituitary gland (23).

PDE4A4, the human analog of rodent PDE4A5, is an isoform expressed in a wide variety of tissues, including lung and various brain regions (18–20, 24, 25). This isoform has UCR1 and UCR2 as well as a unique N-terminal region, which is highly conserved in mammals and has 88% similarity to the N-terminal region of rat PDE4A5 (20). This high degree of conservation between species suggests that the unique amino-terminal region of the PDE4A4 isoform has specific functions (26). The truncation of the PDE4A4 N-terminal region alters its enzymatic activities, its intracellular targeting, and its interaction with other proteins (27, 28). PDE4A5 interacts with AIP and has a reduced expression in *AIP*-mutation-positive adenomas (5, 26, 29, 30). PDE4A4 is expressed in the human pituitary (23). Furthermore, reduced AIP levels were shown to disproportionally enhance the PKA pathway activity under PDE4-specific inhibition in pituitary somatotrophs, pointing out to a link with the disease process involved in Carney complex (31).

By semi quantitative RT-PCR, PDE4C, and PDE4D were also shown to be expressed in the normal pituitary while no expression was detected for PDE8B (14).

## Phosphodiesterases in Pituitary Tumors

Both PDE4A4 and PDE4A8 expression is increased in GH-, PRL-, ACTH- and FSH-secreting adenoma cells compared to their respective normal pituitary cells (23). Interestingly, the augmentation of PDEs observed in pituitary adenomas reflects a consequent increase in PKA activating transcription of cell growth promoting genes, suggesting that these phosphodiesterases might be increased as a possible adaptation or compensation to tumorigenesis, in an attempt to suppress the proliferative drive (23).

Protein-protein interaction between AIP and PDE2A (PDE2A1, PDE2A2, and PDE2A3) has been described (32). Although PDE2As has cGMP as their preferred substrate it may also hydrolyze cAMP (33).

PDE11A has higher expression in GH-secreting adenomas when compared with normal GH-cells (13), which is also described as a phenotype modifier in patients with Carney complex due to *PRKAR1A* mutations (34). The presence and role of PDE11A expression and variants were studied in somatotroph adenomas. Although nonsense and missense PDE11A variants were found in 20% of patients with acromegaly, there was no significant difference in variant frequency compared with controls, suggesting that these variants are unlikely to contribute to the pathogenesis of GH-secreting adenomas since the conservation of the wild-type allele of PDE11A remains in the majority of tumor samples and no significant clinical phenotype could be observed in patients with variant PDE11A (13).

Interestingly, although PDE8B was not detected in normal pituitary, this isoform was shown to be overexpressed in all GH-secreting adenomas, especially higher levels were observed in *gsp*-positive tumors (14). This study also showed that while PDE4C and 4D RNA expression is not increased in *gsp*-mutation negative GH-secreting adenomas compared to normal pituitary, *gsp*-positive samples had seven times higher expression (14). As cAMP-responsive element-binding protein represents the main endpoint of the cAMP pathway, the observed enhanced phosphodiesterase activities may significantly impact the phenotypic expression of *gsp* mutations in somatotrophinomas (14).

## PDE4A Family and AIP

Compared to other PDE isoforms, human PDE4A4 is specifically associated with AIP (also called XAP2 or ARA9), a co-chaperone of HSP90 and HSC70 (26, 35). AIP has several partners, including the aryl hydrocarbon receptor (AhR), PDE4A5, PDE2A, survivin, Tom20, hepatitis B virus protein X, thyroid hormone receptor 1 (TRβ1), Epstein-Barr virus encoded nuclear antigen 3 and peroxisome proliferator-activated receptor—PPARα (36). This gene is described as a tumor suppressor gene in the pituitary (37, 38). Loss-of-function germline mutations predispose to pituitary adenomas, while reduced expression could be lead to altered epigenetic regulation via microRNAs alterations (39, 40).

AIP is expressed in GH- and PRL-cells and electron microscopy studies have identified AIP in the secretory vesicles (5). AIP is abundant in NFPAs (non-functioning pituitary adenomas), and has been shown in corticotrophinomas, although not in the secretory vesicles. However, no AIP expression has been detected in normal gonadotroph and corticotroph cells. Interestingly, it has been demonstrated that the overexpression of wild-type AIP reduces the cell proliferation in three different types of cell lines: GH3 cells, HEK293 cells, and TIG3 fibroblasts (5). These data confirmed that AIP has tumor suppressor gene properties (37, 41). Loss of interaction between AIP and PDE4A5 was seen in a β-galactosidase quantitative two-hybrid assay for pathogenic *AIP* mutations (R81^*^, Q164^*^, K103R, Q217^*^, C238Y, Y248del, R271W, V291M, and R304^*^ (5, 23). For the K241E and R304Q variants, a borderline statistical significance was found for this interaction. For the R16H, V49M, I257V, and A229V variants, there were no clear reduction in their binding (23, 29). Many of the changes disrupting PDE4A5—AIP interaction are known to be important to the stability of the TPR structure of the AIP (42). Clinical data suggest that the R16H, V49M, and A229V may be polymorphisms while the I257V variant affects the TPR structure and clinical data would support a functional impact (29). We summarized data from variants tested in the PDE4A4/5—AIP interaction assay or with PDE4A4 or PDE4A8 immunostaining (Table 1), gathering clinical, frequency, prediction, and experimental data. We note that few variants were tested with more than one functional assay.

**Table 1:** Summary of data for variants tested in in the PDE4A4/5—AIP interaction assays.

**Mutation (DNA level [protein level])**	**Type**	**More than 1 patient**	**Youngest known age of onset**	**Youngest known age of diagnosis**	**Macro**	**Adenoma type**	**Giant**	**Gnomad MAF**	**LOH**	**Frutifly rescued (suggesting not pathogenic) (43)**	**PDE4A5 binding (5, 23, 29)**	**PDE4A4 and/or PDE4A8 staining (30)**	**Half-life (35, 44)**	**HSP90 co-IP (35)**	**Proliferation/cAMP generation (5, 45, 46)**	**Annotation**	**Pathogenic^***$***^**	**References**
c.26G>A (p.R9Q)	M	YES	21	N/A	YES	GH, ACTH, PRL	YES	0,000238	N/A	N/A	N/A	N/A	Normal	N/A	Inc/Inc	Polyphen: benign; SIFT: tolerated	Unlikely	(47–50)
c.38T>A (p.I13N)	M	NO	N/A	N/A	YES	GH	YES	0,00000825	YES	NO	N/A	N/A	N/A	N/A	N/A	Polyphen: possibly_damaging; SIFT: deleterious	Yes	(43, 51)
c.47G>A (p.R16H)	M	YES	N/A	15	YES	GH, PRL	YES	0,00196	NO	YES	< 30%	N/A	Normal	N/A	Inc/Inc	Polyphen: possibly_damaging; SIFT: deleterious	No	(48, 52–63)
c.66_71delAGGAGA (p.G23_E24del)	D	YES	N/A	20	YES	GH	Unknown	Not present	N/A	N/A	high	N/A	N/A	N/A	N/A	–	Yes	(53)
c.145G>A (V49M)	M	NO	N/A	N/A	N/A	GH	YES	0,0002745	NO	N/A	>30%	N/A	Short	N/A	Inc/Inc	Polyphen: benign; SIFT: deleterious	Unlikely	(64)
c.208C>A (p.L70M)	M	YES	N/A	22	YES	GH & PRL, PRL	Unknown	Not present	N/A	N/A	< 30%	N/A	N/A	N/A	N/A	Polyphen: probably_damaging; SIFT: deleterious	Likely	(65)
c.241C>T (p.R81*)	N	YES	3	4	YES	GH	YES	Not present	YES	N/A	< 30%	N/A	N/A	N/A	N/A	Loss-of-Function: High-confidence	Yes	(5, 66–69)
c.308A>G (p.K103R)	M	NO	N/A	6	YES	ACTH	NO	0,0000041	N/A	N/A	>30%	N/A	Normal	N/A	no change/no change	Polyphen: benign; SIFT: tolerated	VUS	(46, 70)
c.490C>T (p.Q164*)	N	YES	15	23	YES	GH	YES	Not present	N/A	N/A	< 30%	low	N/A	N/A	N/A	Loss-of-Function: High-confidence	Yes	(29)
c.562C>T (R188W)	M	NO	10	12	YES	GH & PRL	YES	0,000032	N/A	N/A	N/A	N/A	Very short	N/A	N/A	Polyphen: benign; SIFT: deleterious	Likely	(35)
c.649C>T (p.Q217*)	N	YES	N/A	17	YES	GH, GH & PRL	Unknown	Not present	N/A	N/A	< 30%	N/A	N/A	N/A	N/A	Loss-of-Function: High-confidence	Yes	(55, 71)
c.662dupC (p.E222*)	F	NO	22	24	YES	GH	YES	Not present	N/A	N/A	< 30%	low	N/A	N/A	N/A	Loss-of-Function: High-confidence	Yes	(29)
c.713G>A (p.C238Y)	M	YES	18	19	YES	GH	NO	0,000004025	YES	NO	< 30%	N/A	Very short	N/A	Inc / N/A	Polyphen: possibly_damaging; SIFT: deleterious	Yes	(5, 23, 35)
c.721A>G (p.K241E)	M	YES	N/A	40	YES	PRL, NFPA	NO	0,00003622	N/A	N/A	< 30%	N/A	Short	N/A	N/A	Polyphen: possibly_damaging; SIFT: tolerated	Likely	(55)
c.742_744delTAC (p.Y248del)	D	NO	N/A	19	YES	GH	YES	Not present	YES	N/A	<30%	N/A	N/A	N/A	N/A	Loss-of-Function: High confidence	Yes	(72)
c.760T>C (p.C254R)	M	YES	14	33	YES	GH	YES	Not present	N/A	N/A	N/A	N/A	Very short	N/A	N/A	Polyphen: possibly_damaging; SIFT: tolerated	Likely	(35)
c.769A>G (p.I257V)	M	NO	N/A	39	YES	TSH	NO	Not present	N/A	N/A	<30%	N/A	Short	lost	N/A	Polyphen: benign; SIFT: tolerated	Likely	(73)
c.805_825dup (F269_H275dup)	I	YES	9	13	YES	GH, GH & PRL	YES	Not present	N/A	N/A	N/A	low	Very short	N/A	N/A	Loss-of-Function: High-confidence	Yes	(5, 44)
c.811C>T (p.R271W)	M	NO	15	16	YES	GH & PRL, GH, PRL	YES	Not present	N/A	N/A	<30%	N/A	Very short	N/A	N/A	Polyphen: probably_damaging; SIFT: deleterious	Yes	(5, 29, 55, 59, 68, 69, 74, 75)
c.815G>A (p.G272D)	M	YES	38	43	YES	GH	YES	0,00000409	N/A	NO	<30%	N/A	N/A	lost	N/A	Polyphen: possibly_damaging; SIFT: deleterious	Likely	(76–78)
c.871G>A (p.V291M)	M	NO	N/A	30	N/A	GH & PRL	NO	Not present	NO	N/A	<30%	N/A	Very short	N/A	N/A	Polyphen: probably_damaging; SIFT: deleterious	Likely	(48)
c.910C>T (p.R304*)	N	YES	6	6	YES	GH, PRL, GH & PRL	YES	0,00001436	YES	N/A	<30%	low	Very short	N/A	N/A	Loss-of-Function: High-confidence	Yes	(5, 29, 37, 48, 54, 55, 59, 79–84)
c.911G>A (p.R304Q)	M	YES	17	18	YES	GH, PRL, GH & PRL, ACTH	YES	0,00000842	NO	YES	<30%	N/A	Normal	N/A	N/A	Polyphen: possibly_damaging; SIFT: tolerated	VUS	(5, 29, 48, 53, 54, 59, 81, 85–88)
c.911C>T (p.R304*)	N	YES	6	6	YES	GH, GH & PRL	YES	0.00001436	YES	N/A	N/A	low N/A	Very short	lost	N/A	Loss-of-Function: High-confidence	Yes	(37 and many others)
c.940C>T (p.R314W)	M	NO	18	N/A	YES	GH	YES	0,00001445	YES	YES	N/A	N/A	N/A	N/A	N/A	Polyphen: probably_damaging; SIFT: deleterious	VUS	(60)
c.991T>C (p.*331R)	M	NO	11	15	YES	GH	YES	Not present	YES	N/A	N/A	N/A	N/A	N/A	N/A	Loss of stop codon	Likely	(89)

*M, missense; N, nonsense; D, deletion; N/A, not available; LOH, loss of heterozygosity; $ Pathogenicity is based on authors' judgement following the American College of Medical Genetics and Genomics principles (90); F, frameshift; I, insertion; MAF, minor allele frequency; Inc, increased; VUS, variant of unknown significance*.

There are different PDE4A4 and PDE4A8 expression patterns in somatotroph adenomas from patients with *AIP* mutations compared to patients with wild-type *AIP* (Table 2). It has been previously shown that the C-terminal part of AIP is a key for its functional effects. Mutations affecting the C-terminal end lead either to nonsense-mediated decay of the abnormal RNA (probably relevant for p.E222^*^), create a protein with significantly shortened half-life [as shown for p.F269_H275dup (44) and p.R304^*^ (35)], or lose interaction with protein partners (91). *AIP* mutation-positive samples had significantly decreased PDE4A4 expression compared to sporadic somatotroph adenomas, suggesting that *AIP* mutation-positive somatotroph cells are unable to upregulate PDE4A4 expression (30).

**Table 2:** Phosphodiesterases (PDE) isoforms and their respective protein/RNA expression in different pituitary cells types.

	**Normal pituitary**	**Sporadic GH-secreting Adenomas**	**Sporadic PRL- secreting adenomas**	**Sporadic ACTH- secreting Adenomas**	**Sporadic Non-functioning adenomas (FSH+)**	**GH-secreting adenoma AIP mutation**
PDE4A (14)	Presence of RNA in GH cells	Gsp+ RNA ↑ Gsp– RNA =	NA	NA	NA	NA
PDE4A4 (23, 30)	Presence of the protein in GH/PRL/ACTH/FSH cells	↑	↑	↑	↑	F269_H275dup = R304* = E222* =
PDE4A8 (23, 30)	Presence of the protein in GH/PRL/ACTH/FSH cells	↑	↑	↑	↑	F269_H275dup ↓ R304* = Q164* ↓
PDE4B (14)	Presence of RNA in GH cells	Gsp+ RNA ↑ Gsp- RNA ↑	NA	NA	NA	NA
PDE4C (14)	Presence of RNA in GH cells	Gsp+ RNA ↑ Gsp- RNA =	NA	NA	NA	NA
PDE4D (14)	Presence of RNA in GH cells	Gsp+ RNA ↑ Gsp- RNA =	NA	NA	NA	NA
PDE8B (14)	Absent GH cells	Gsp+ RNA ↑ Gsp- RNA ↑	NA	NA	NA	NA
PDE11A (13)	Presence of the protein GH cells	Protein ↑	NA	NA	NA	NA

*All comparisons are in relation to the respective normal cell. ↑ increase ↓ decrease = equal*.

For PDE4A8, although no interaction with AIP has been shown due to the fact that this protein cannot be produced *in vitro* for the two-hybrid assay, a reduced protein expression was observed in *AIP* mutation-positive samples (30). These differences in PDE4A8 protein expression suggest that, similarly to PDE4A4/5, AIP may support expression or stability of PDE4A8, leading to closely regulated cAMP pathway activity (30).

## Phosphodiesterase Inhibition

The use of PDEs inhibitors, either selective or nonselective, represents an effective targeted strategy for the treatment of many human diseases, such as respiratory disorders, erectile dysfunction, prostate cancer and inflammatory diseases (92–95).

The inhibitory effect of heterologously expressed *AIP* on cAMP levels has not been altered by the general inhibition of phosphodiesterases (by IBMX) or the PDE4-specific inhibitor rolipram. Furthermore, the GH secretion was not altered by the use of these inhibitors (45). However, it has been shown that in rat somatotrophinoma GH3 cells, AIP regulates cAMP signaling and GH secretion independently of the AIP–PDE interaction. In the rat somatotrophinoma GH3 cells treated with forskolin, a drug that increases the cAMP levels, it was shown that the *AIP* overexpression could attenuate the cAMP response to the drug, even in the absence of PDE activity, while *AIP* knockdown activates the cAMP pathway. Although these effects are not observed in untreated cells, these results suggest that AIP may itself act as a tumor suppressor by reducing cAMP signaling (38, 45). However, GH-secreting adenomas with positive *AIP* mutation show reduced phosphorylation of the mitogen-activated protein kinases (MAPKs) 3 and 1 as well as reduction of phosphorylation of the cAMP response element binding protein (CREB). Also, AIP knockout causes reduced CREB phosphorylation in mouse embryonic fibroblasts although AIP knockdown rat somatotrophinoma GH3 cells do not show any of these changes on cAMP effectors (38, 96). To this point, the binding between PDE4A5-AIP does not seem to be the only regulator of this pathway.

In rat corticotroph cells, cAMP levels are related to selective activity of PDE1 (PDE1A or PDE1C) or PDE4, depending on the type and intensity of stress conditions (97). On the other hand, mouse corticotroph cell line AtT-20 with forskolin-induced elevated cAMP levels showed no response to IBMX with or without rolipram (98). Further studies are needed to clarify the possible therapeutic role of PDE manipulation in pituitary adenomas.

## Summary

The cAMP pathway plays a key role in somatotroph tumorigenesis, as suggested by altered cAMP pathway in *GNAS, PRKAR1A, AIP*, and *GPR101* mutated samples. Targeted therapies influencing this pathway may have a key role in the medical treatment of these currently often treatment-resistant conditions.

## Author Contributions

All authors listed have made a substantial, direct and intellectual contribution to the work, and approved it for publication.

### Conflict of Interest Statement

The authors declare that the research was conducted in the absence of any commercial or financial relationships that could be construed as a potential conflict of interest.
